# (*Z*)-2-Amino-5-[2,4-dimeth­oxy-6-(4-methoxy­styr­yl)benzyl­idene]-1,3-thia­zol-4(5*H*)-one methanol solvate

**DOI:** 10.1107/S1600536810018520

**Published:** 2010-06-26

**Authors:** Nikhil Reddy Madadi, Thirupathi Reddy Yerram Reddy, Narsimha Reddy Penthala, Sean Parkin, Peter A. Crooks

**Affiliations:** a Department of Pharmaceutical Sciences, College of Pharmacy, University of Kentucky, Lexington, KY 40536, USA; b Department of Chemistry, University of Kentucky, Lexington, KY 40506, USA

## Abstract

In the crystal structure of the title compound, C_21_H_20_N_2_O_4_S·CH_3_OH, mol­ecules are linked into chains by a series of inter­molecular N—H⋯O, N—H⋯N and O—H⋯O hydrogen bonds. The mol­ecular structure shows a double bond with *Z* geometry, connecting the thia­zolone and resveratrol units. The dihedral angle between the thiazolone ring and the nearest dimethoxy­benzene ring is 53.02 (7)°.

## Related literature

For related structure–activitystudies, see; Aggarwal *et al.* (2004 ▶); Pettit *et al.* (1995 ▶); Cushman *et al.* (1991 ▶).
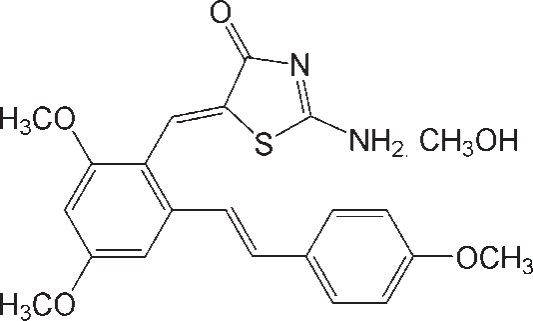


## Experimental

### 

#### Crystal data

C_21_H_20_N_2_O_4_S·CH_4_O
*M* *_r_* = 428.49
Monoclinic, 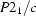

*a* = 10.6243 (2) Å
*b* = 22.2530 (5) Å
*c* = 9.0562 (2) Å
β = 93.028 (1)°
*V* = 2138.10 (8) Å^3^
*Z* = 4
Cu *K*α radiation
μ = 1.65 mm^−1^
*T* = 90 K
0.15 × 0.08 × 0.02 mm

#### Data collection

Bruker X8 Proteum diffractometer
Absorption correction: multi-scan (*SADABS*; Bruker, 2006 ▶) *T*_min_ = 0.777, *T*_max_ = 0.968
31098 measured reflections
3911 independent reflections
3631 reflections with *I* > 2σ(*I*)
*R*_int_ = 0.044

#### Refinement

*R*[*F* ^2^ > 2σ(*F* ^2^)] = 0.043
*wR*(*F* ^2^) = 0.112
*S* = 1.13
3911 reflections
276 parameters
H-atom parameters constrained
Δρ_max_ = 0.51 e Å^−3^
Δρ_min_ = −0.30 e Å^−3^

### 

Data collection: *APEX2* (Bruker, 2006 ▶); cell refinement: *SAINT* (Bruker, 2006 ▶); data reduction: *SAINT*; program(s) used to solve structure: *SHELXS97* (Sheldrick, 2008 ▶); program(s) used to refine structure: *SHELXL97* (Sheldrick, 2008 ▶); molecular graphics: *XP* in *SHELXTL* (Sheldrick, 2008 ▶); software used to prepare material for publication: *SHELXL97* and local procedures.

## Supplementary Material

Crystal structure: contains datablocks global, I. DOI: 10.1107/S1600536810018520/fj2286sup1.cif

Structure factors: contains datablocks I. DOI: 10.1107/S1600536810018520/fj2286Isup2.hkl

Additional supplementary materials:  crystallographic information; 3D view; checkCIF report

## Figures and Tables

**Table 1:** Hydrogen-bond geometry (Å, °)

*D*—H⋯*A*	*D*—H	H⋯*A*	*D*⋯*A*	*D*—H⋯*A*
N2—H2*A*⋯O4^i^	0.88	2.07	2.926 (2)	163
N2—H2*A*⋯N1^i^	0.88	2.64	3.175 (2)	120
N2—H2*B*⋯O1*S*^ii^	0.88	2.05	2.872 (2)	154
O1*S*—H1*S*⋯O4	0.84	1.88	2.716 (2)	172

Symmetry codes: (i) 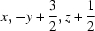; (ii) 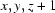.

**Fig. 1.**
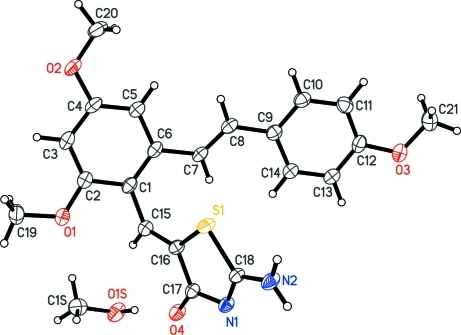
A view of the molecule with the atom numbering scheme. Displacement ellipsoids are drawn at the 50% probability level.

## supplementary crystallographic information

### Comment

Many natural products possessing a trimethoxybenzene ring, e.g., colchicines, and podophyllotoxins, are potent cytotoxic agents and exert their antitumor properties by their antitubulin activity. In view of the activity of such trimethoxybenzenes, similar structurally related stilbene moieties have been studied. The trihydroxy compound, resveratrol, a naturally occurring phytoalexin (trans-3, 4, 5-trihydroxystilbene) present in grapes, berries, peanuts,and red wine [Aggarwal *et al.*,2004, Pettit *et al.*, 1995) is reported to be a potential cancer chemotherapeutic agent based on its striking inhibitory effects on cellular events associated with cancer initiation, promotion, and progression. (Cushman *et al.*, 1991). These observations encouraged us to design and synthesise a series of novel trimethoxy resveratrol analogs that were expected to function as potent cytotoxic agents against lung and breast cancer cells. The structural characterization of the title compound by x-ray analysis was performed to determine the geometry (*E* vs *Z*) of the double bond connecting the thiozolone ring and the resveratrol moiety, which cannot be easily determined by NMR spectroscopic analysis, and to obtain detailed information on the structural conformation of the molecule, that may be useful in structure-activity relationship (SAR) analysis. The title compound was synthesized in two steps. In step one, the formylation of (*E*)-1, 3-dimethoxy-5- (4-methoxystyryl)benzene with a slight excess of phosphorous oxychloride in dimethylformamide at 0 °C resulted the formation of trans-2-formyl-3, 4', 5-trimethoxystilbene. In step two, the reaction of trans-2-formyl-3, 4', 5-trimethoxystilbene with the active methelene compound, 2-aminothiazol-4(*5H*)-one in presence of ammonium acetate in acetic acid under microwave irradiation conditions yielded the title compound, (*Z*)-2-amino-5-[2,4-dimethoxy-6- (4-methoxystyryl)benzylidene]thiazol-4(*5H*)-one in 90% yield. The x-ray analysis studies revealed that the double bond connecting the thiazolone and resveratrol moieties has the *Z* geometry. The dihedral angle between the plane of the thiazolone ring and the plane of the nearest phenyl ring is 53.02 (7)°. The crystal packing is stabilized by a series of N—H···O, N—H···N and O—H···O intermolecular hydrogen bonds.

### Experimental

A mixture of trans-2-formyl-3,4',5-trimethoxystilbene (50 mg, 1 mmol), 2-aminothiazol-4(5*H*)-one (20.44 mg, 1.1 mmol), ammonium acetate (13.56 mg, 1.1 mmol) and acetic acid (0.25 ml) was irradiated in a domestic microwave oven for 60 sec with intermittent cooling to room temperature every 20 sec. The reaction mixture was allowed to cool to room temperature, and treated with saturated aqueous sodium bicarbonate solution. The precipitate thus obtained was collected by filtration, washed with cold water and dried, to afford the crude product. Crystallization from methanol gave a white crystalline product of (*Z*)-2-amino-5-[2,4-dimethoxy-6-(4-methoxystyryl) benzylidene]thiazol-4(5*H*)-one methanolate, which was suitable for x-ray analysis. ^1^H NMR (DMSO-d_6_): δ 3.77 (*s*, 3H, -OCH_3_), 3.82 (*s*, 3H, -OCH_3_), 3.86 (*s*, 3H, -OCH_3_), 6.54-6.55 (*d*, *J*=2 Hz, 1H), 6.90-6.91 (*m*, 1H), 6.93-6.95 (*d*, *J*=2 Hz, 3H), 7.20-7.23 (*d*, *J*=16 Hz, 1H), 7.47-7.49 (*d*, *J*=9 Hz, 2H), 7.61 (*s*, 1H), 8.83 (*s*, 1H), 9.12 (*s*, 1H) ppm. ^13^C NMR (DMSO-d_6_): δ 55.6, 55.9, 56.3, 98.1, 102.8, 114.9, 115.9, 124.2,125.7, 128.6, 130.2, 131.6, 134.6, 138.4, 150.5, 158.9, 159.9, 161.6, 176.6, 180.3, 181.3. M. P: 172-175 °C

### Refinement

H atoms were found in difference Fourier maps and subsequently placed in idealized positions with constrained distances of 0.98 Å (RCH_3_), 0.95 Å (C_Ar_H), and with U_iso_(H) values set to either 1.2U_eq_ or 1.5U_eq_ (RCH_3_, OH) of the attached atom.

### Crystal data

**Table:**

C_21_H_20_N_2_O_4_S·CH_4_O	*F*(000) = 904
*M**_r_* = 428.49	*D*_x_ = 1.331 Mg m^−^^3^
Monoclinic, *P*2_1_/*c*	Cu *K*α radiation, λ = 1.54178 Å
Hall symbol: -P 2ybc	Cell parameters from 9054 reflections
*a* = 10.6243 (2) Å	θ = 4.0–68.4°
*b* = 22.2530 (5) Å	µ = 1.65 mm^−^^1^
*c* = 9.0562 (2) Å	*T* = 90 K
β = 93.028 (1)°	Lath, yellow
*V* = 2138.10 (8) Å^3^	0.15 × 0.08 × 0.02 mm
*Z* = 4	

### Data collection

**Table:**

Bruker X8 Proteum diffractometer	3911 independent reflections
Radiation source: fine-focus rotating anode	3631 reflections with *I* > 2σ(*I*)
graded multilayer optics	*R*_int_ = 0.044
Detector resolution: 5.6 pixels mm^-1^	θ_max_ = 68.4°, θ_min_ = 4.0°
φ and ω scans	*h* = −12→12
Absorption correction: multi-scan (*SADABS*; Bruker, 2006)	*k* = −26→26
*T*_min_ = 0.777, *T*_max_ = 0.968	*l* = −10→10
31098 measured reflections	

### Refinement

**Table:**

Refinement on *F*^2^	Primary atom site location: structure-invariant direct methods
Least-squares matrix: full	Secondary atom site location: difference Fourier map
*R*[*F*^2^ > 2σ(*F*^2^)] = 0.043	Hydrogen site location: inferred from neighbouring sites
*wR*(*F*^2^) = 0.112	H-atom parameters constrained
*S* = 1.13	*w* = 1/[σ^2^(*F*_o_^2^) + (0.0357*P*)^2^ + 2.3067*P*] where *P* = (*F*_o_^2^ + 2*F*_c_^2^)/3
3911 reflections	(Δ/σ)_max_ < 0.001
276 parameters	Δρ_max_ = 0.51 e Å^−^^3^
0 restraints	Δρ_min_ = −0.30 e Å^−^^3^

### Special details

**Table:**

Geometry. All esds (except the esd in the dihedral angle between two l.s. planes) are estimated using the full covariance matrix. The cell esds are taken into account individually in the estimation of esds in distances, angles and torsion angles; correlations between esds in cell parameters are only used when they are defined by crystal symmetry. An approximate (isotropic) treatment of cell esds is used for estimating esds involving l.s. planes.
Refinement. Refinement of *F*^2^ against ALL reflections. The weighted *R*-factor wR and goodness of fit *S* are based on *F*^2^, conventional *R*-factors *R* are based on *F*, with *F* set to zero for negative *F*^2^. The threshold expression of *F*^2^ > 2σ(*F*^2^) is used only for calculating *R*-factors(gt) etc. and is not relevant to the choice of reflections for refinement. *R*-factors based on *F*^2^ are statistically about twice as large as those based on *F*, and *R*- factors based on ALL data will be even larger.

### Fractional atomic coordinates and isotropic or equivalent isotropic displacement parameters (Å^2^)

**Table:**

	*x*	*y*	*z*	*U*_iso_*/*U*_eq_	
S1	0.73380 (5)	0.59495 (2)	0.83763 (5)	0.02575 (15)	
O1	0.84106 (14)	0.45171 (6)	0.46089 (17)	0.0294 (3)	
N1	0.79198 (16)	0.69625 (7)	0.70367 (18)	0.0233 (4)	
C1	0.67303 (19)	0.48654 (9)	0.5941 (2)	0.0230 (4)	
O2	0.56944 (16)	0.31027 (7)	0.6733 (2)	0.0401 (4)	
N2	0.78786 (18)	0.70133 (8)	0.95934 (19)	0.0288 (4)	
H2A	0.8067	0.7398	0.9591	0.035*	
H2B	0.7766	0.6828	1.0435	0.035*	
C2	0.73882 (19)	0.43671 (9)	0.5382 (2)	0.0247 (4)	
O3	0.15908 (16)	0.72923 (7)	1.04700 (19)	0.0379 (4)	
C3	0.7014 (2)	0.37874 (9)	0.5646 (2)	0.0287 (5)	
H3	0.7459	0.3457	0.5260	0.034*	
O4	0.79309 (15)	0.66720 (6)	0.46217 (15)	0.0276 (3)	
C4	0.5968 (2)	0.36913 (9)	0.6495 (2)	0.0285 (5)	
C5	0.5283 (2)	0.41643 (9)	0.7024 (2)	0.0254 (4)	
H5	0.4560	0.4090	0.7571	0.031*	
C6	0.56614 (19)	0.47574 (9)	0.6748 (2)	0.0227 (4)	
C7	0.49002 (19)	0.52638 (9)	0.7237 (2)	0.0230 (4)	
H7	0.4958	0.5629	0.6702	0.028*	
C8	0.41358 (19)	0.52599 (9)	0.8360 (2)	0.0252 (4)	
H8	0.4032	0.4890	0.8861	0.030*	
C9	0.34427 (19)	0.57835 (9)	0.8881 (2)	0.0249 (4)	
C10	0.2585 (2)	0.57198 (10)	0.9977 (2)	0.0280 (5)	
H10	0.2434	0.5330	1.0359	0.034*	
C11	0.1938 (2)	0.62079 (10)	1.0535 (2)	0.0296 (5)	
H11	0.1356	0.6150	1.1283	0.036*	
C12	0.2153 (2)	0.67762 (10)	0.9992 (2)	0.0296 (5)	
C13	0.2998 (2)	0.68532 (10)	0.8890 (3)	0.0363 (5)	
H13	0.3138	0.7243	0.8502	0.044*	
C14	0.3634 (2)	0.63662 (10)	0.8357 (3)	0.0325 (5)	
H14	0.4219	0.6428	0.7614	0.039*	
C15	0.71662 (18)	0.54678 (9)	0.5548 (2)	0.0223 (4)	
H15	0.7306	0.5531	0.4533	0.027*	
C16	0.73910 (19)	0.59395 (9)	0.6445 (2)	0.0224 (4)	
C17	0.77662 (18)	0.65520 (9)	0.5932 (2)	0.0218 (4)	
C18	0.77655 (19)	0.67159 (9)	0.8345 (2)	0.0227 (4)	
C19	0.9131 (2)	0.40406 (10)	0.4010 (3)	0.0352 (5)	
H19A	0.9469	0.3783	0.4815	0.053*	
H19B	0.9829	0.4211	0.3480	0.053*	
H19C	0.8590	0.3802	0.3326	0.053*	
C20	0.4740 (2)	0.29656 (11)	0.7742 (3)	0.0427 (6)	
H20A	0.4925	0.3177	0.8678	0.064*	
H20B	0.4726	0.2531	0.7921	0.064*	
H20C	0.3917	0.3095	0.7316	0.064*	
C21	0.0789 (2)	0.72365 (12)	1.1677 (3)	0.0387 (6)	
H21A	0.0098	0.6959	1.1407	0.058*	
H21B	0.0443	0.7631	1.1909	0.058*	
H21C	0.1276	0.7081	1.2543	0.058*	
O1S	0.80228 (17)	0.61397 (7)	0.19308 (17)	0.0374 (4)	
H1S	0.7956	0.6277	0.2787	0.056*	
C1S	0.8883 (2)	0.56477 (11)	0.1984 (3)	0.0355 (5)	
H1S1	0.9573	0.5731	0.2717	0.053*	
H1S2	0.9226	0.5593	0.1009	0.053*	
H1S3	0.8442	0.5281	0.2262	0.053*	

### Atomic displacement parameters (Å^2^)

**Table:**

	*U*^11^	*U*^22^	*U*^33^	*U*^12^	*U*^13^	*U*^23^
S1	0.0407 (3)	0.0184 (3)	0.0178 (3)	−0.0068 (2)	−0.0021 (2)	0.00092 (18)
O1	0.0325 (8)	0.0229 (7)	0.0330 (8)	0.0030 (6)	0.0050 (6)	−0.0030 (6)
N1	0.0327 (9)	0.0184 (8)	0.0185 (8)	−0.0006 (7)	−0.0005 (7)	0.0005 (6)
C1	0.0286 (10)	0.0183 (10)	0.0215 (10)	0.0002 (8)	−0.0055 (8)	−0.0022 (8)
O2	0.0421 (9)	0.0152 (7)	0.0638 (12)	−0.0006 (6)	0.0108 (8)	0.0004 (7)
N2	0.0457 (11)	0.0208 (9)	0.0196 (9)	−0.0054 (8)	−0.0004 (8)	−0.0007 (7)
C2	0.0267 (10)	0.0236 (10)	0.0232 (10)	0.0020 (8)	−0.0036 (8)	−0.0015 (8)
O3	0.0432 (9)	0.0266 (8)	0.0448 (10)	0.0034 (7)	0.0121 (7)	−0.0054 (7)
C3	0.0316 (11)	0.0203 (10)	0.0337 (12)	0.0045 (8)	−0.0033 (9)	−0.0026 (9)
O4	0.0431 (9)	0.0214 (7)	0.0185 (7)	−0.0005 (6)	0.0018 (6)	0.0010 (5)
C4	0.0326 (11)	0.0160 (10)	0.0363 (12)	−0.0020 (8)	−0.0043 (9)	0.0008 (8)
C5	0.0273 (10)	0.0201 (10)	0.0284 (11)	−0.0008 (8)	−0.0032 (8)	−0.0001 (8)
C6	0.0276 (10)	0.0184 (9)	0.0211 (10)	0.0008 (8)	−0.0071 (8)	−0.0023 (8)
C7	0.0264 (10)	0.0162 (9)	0.0256 (11)	−0.0015 (8)	−0.0059 (8)	−0.0007 (8)
C8	0.0295 (11)	0.0190 (10)	0.0263 (11)	−0.0028 (8)	−0.0047 (8)	0.0000 (8)
C9	0.0279 (10)	0.0236 (10)	0.0227 (10)	−0.0024 (8)	−0.0028 (8)	−0.0019 (8)
C10	0.0356 (11)	0.0236 (11)	0.0244 (11)	−0.0031 (9)	−0.0015 (9)	0.0018 (8)
C11	0.0312 (11)	0.0348 (12)	0.0230 (11)	−0.0035 (9)	0.0022 (8)	−0.0023 (9)
C12	0.0322 (11)	0.0238 (11)	0.0325 (12)	0.0008 (9)	−0.0002 (9)	−0.0076 (9)
C13	0.0440 (13)	0.0215 (11)	0.0446 (14)	−0.0035 (10)	0.0121 (11)	−0.0015 (10)
C14	0.0364 (12)	0.0229 (11)	0.0390 (13)	−0.0042 (9)	0.0098 (10)	−0.0040 (9)
C15	0.0259 (10)	0.0215 (10)	0.0192 (10)	0.0014 (8)	−0.0020 (8)	0.0012 (8)
C16	0.0249 (10)	0.0188 (10)	0.0232 (10)	−0.0005 (8)	−0.0015 (8)	0.0023 (8)
C17	0.0252 (10)	0.0195 (10)	0.0206 (10)	0.0012 (8)	−0.0010 (8)	0.0011 (8)
C18	0.0258 (10)	0.0186 (9)	0.0234 (10)	−0.0023 (8)	−0.0001 (8)	−0.0017 (8)
C19	0.0337 (12)	0.0319 (12)	0.0401 (13)	0.0067 (10)	0.0038 (10)	−0.0053 (10)
C20	0.0417 (14)	0.0210 (11)	0.0662 (18)	−0.0025 (10)	0.0092 (12)	0.0090 (11)
C21	0.0366 (13)	0.0387 (13)	0.0413 (14)	0.0073 (10)	0.0058 (10)	−0.0064 (11)
O1S	0.0644 (11)	0.0277 (8)	0.0199 (8)	0.0013 (8)	0.0020 (7)	0.0003 (6)
C1S	0.0409 (13)	0.0380 (13)	0.0275 (12)	−0.0060 (10)	0.0017 (10)	−0.0014 (10)

### Geometric parameters (Å, °)

**Table:**

S1—C16	1.753 (2)	C9—C10	1.390 (3)
S1—C18	1.765 (2)	C9—C14	1.399 (3)
O1—C2	1.365 (3)	C10—C11	1.394 (3)
O1—C19	1.431 (3)	C10—H10	0.9500
N1—C18	1.324 (3)	C11—C12	1.380 (3)
N1—C17	1.358 (3)	C11—H11	0.9500
C1—C6	1.403 (3)	C12—C13	1.388 (3)
C1—C2	1.418 (3)	C13—C14	1.378 (3)
C1—C15	1.468 (3)	C13—H13	0.9500
O2—C4	1.362 (3)	C14—H14	0.9500
O2—C20	1.433 (3)	C15—C16	1.341 (3)
N2—C18	1.310 (3)	C15—H15	0.9500
N2—H2A	0.8800	C16—C17	1.501 (3)
N2—H2B	0.8800	C19—H19A	0.9800
C2—C3	1.375 (3)	C19—H19B	0.9800
O3—C12	1.375 (3)	C19—H19C	0.9800
O3—C21	1.426 (3)	C20—H20A	0.9800
C3—C4	1.401 (3)	C20—H20B	0.9800
C3—H3	0.9500	C20—H20C	0.9800
O4—C17	1.237 (2)	C21—H21A	0.9800
C4—C5	1.380 (3)	C21—H21B	0.9800
C5—C6	1.406 (3)	C21—H21C	0.9800
C5—H5	0.9500	O1S—C1S	1.425 (3)
C6—C7	1.469 (3)	O1S—H1S	0.8400
C7—C8	1.334 (3)	C1S—H1S1	0.9800
C7—H7	0.9500	C1S—H1S2	0.9800
C8—C9	1.470 (3)	C1S—H1S3	0.9800
C8—H8	0.9500		
			
C16—S1—C18	88.54 (9)	C14—C13—C12	120.2 (2)
C2—O1—C19	118.01 (17)	C14—C13—H13	119.9
C18—N1—C17	111.41 (17)	C12—C13—H13	119.9
C6—C1—C2	118.67 (18)	C13—C14—C9	121.8 (2)
C6—C1—C15	123.84 (18)	C13—C14—H14	119.1
C2—C1—C15	117.36 (18)	C9—C14—H14	119.1
C4—O2—C20	118.05 (18)	C16—C15—C1	128.05 (19)
C18—N2—H2A	120.0	C16—C15—H15	116.0
C18—N2—H2B	120.0	C1—C15—H15	116.0
H2A—N2—H2B	120.0	C15—C16—C17	124.40 (18)
O1—C2—C3	124.34 (19)	C15—C16—S1	126.89 (16)
O1—C2—C1	114.37 (18)	C17—C16—S1	108.69 (14)
C3—C2—C1	121.28 (19)	O4—C17—N1	122.94 (18)
C12—O3—C21	117.11 (18)	O4—C17—C16	123.11 (18)
C2—C3—C4	118.93 (19)	N1—C17—C16	113.95 (17)
C2—C3—H3	120.5	N2—C18—N1	123.57 (18)
C4—C3—H3	120.5	N2—C18—S1	119.09 (15)
O2—C4—C5	123.9 (2)	N1—C18—S1	117.32 (15)
O2—C4—C3	114.60 (19)	O1—C19—H19A	109.5
C5—C4—C3	121.50 (19)	O1—C19—H19B	109.5
C4—C5—C6	119.6 (2)	H19A—C19—H19B	109.5
C4—C5—H5	120.2	O1—C19—H19C	109.5
C6—C5—H5	120.2	H19A—C19—H19C	109.5
C1—C6—C5	120.01 (18)	H19B—C19—H19C	109.5
C1—C6—C7	119.95 (18)	O2—C20—H20A	109.5
C5—C6—C7	119.97 (19)	O2—C20—H20B	109.5
C8—C7—C6	126.26 (19)	H20A—C20—H20B	109.5
C8—C7—H7	116.9	O2—C20—H20C	109.5
C6—C7—H7	116.9	H20A—C20—H20C	109.5
C7—C8—C9	125.10 (19)	H20B—C20—H20C	109.5
C7—C8—H8	117.5	O3—C21—H21A	109.5
C9—C8—H8	117.5	O3—C21—H21B	109.5
C10—C9—C14	116.7 (2)	H21A—C21—H21B	109.5
C10—C9—C8	120.47 (19)	O3—C21—H21C	109.5
C14—C9—C8	122.80 (19)	H21A—C21—H21C	109.5
C9—C10—C11	122.3 (2)	H21B—C21—H21C	109.5
C9—C10—H10	118.9	C1S—O1S—H1S	109.5
C11—C10—H10	118.9	O1S—C1S—H1S1	109.5
C12—C11—C10	119.4 (2)	O1S—C1S—H1S2	109.5
C12—C11—H11	120.3	H1S1—C1S—H1S2	109.5
C10—C11—H11	120.3	O1S—C1S—H1S3	109.5
O3—C12—C11	124.8 (2)	H1S1—C1S—H1S3	109.5
O3—C12—C13	115.6 (2)	H1S2—C1S—H1S3	109.5
C11—C12—C13	119.7 (2)		
			
C19—O1—C2—C3	1.1 (3)	C9—C10—C11—C12	−0.2 (3)
C19—O1—C2—C1	179.79 (18)	C21—O3—C12—C11	4.0 (3)
C6—C1—C2—O1	179.80 (17)	C21—O3—C12—C13	−175.4 (2)
C15—C1—C2—O1	3.9 (3)	C10—C11—C12—O3	−178.8 (2)
C6—C1—C2—C3	−1.5 (3)	C10—C11—C12—C13	0.6 (3)
C15—C1—C2—C3	−177.44 (19)	O3—C12—C13—C14	178.5 (2)
O1—C2—C3—C4	178.09 (19)	C11—C12—C13—C14	−1.0 (4)
C1—C2—C3—C4	−0.5 (3)	C12—C13—C14—C9	1.0 (4)
C20—O2—C4—C5	−8.1 (3)	C10—C9—C14—C13	−0.5 (3)
C20—O2—C4—C3	172.3 (2)	C8—C9—C14—C13	−178.2 (2)
C2—C3—C4—O2	−178.10 (19)	C6—C1—C15—C16	52.1 (3)
C2—C3—C4—C5	2.3 (3)	C2—C1—C15—C16	−132.2 (2)
O2—C4—C5—C6	178.4 (2)	C1—C15—C16—C17	−176.34 (19)
C3—C4—C5—C6	−2.0 (3)	C1—C15—C16—S1	5.2 (3)
C2—C1—C6—C5	1.8 (3)	C18—S1—C16—C15	179.4 (2)
C15—C1—C6—C5	177.43 (19)	C18—S1—C16—C17	0.77 (14)
C2—C1—C6—C7	−175.00 (18)	C18—N1—C17—O4	−176.10 (19)
C15—C1—C6—C7	0.7 (3)	C18—N1—C17—C16	3.4 (2)
C4—C5—C6—C1	−0.1 (3)	C15—C16—C17—O4	−1.8 (3)
C4—C5—C6—C7	176.70 (19)	S1—C16—C17—O4	176.94 (16)
C1—C6—C7—C8	−157.3 (2)	C15—C16—C17—N1	178.79 (19)
C5—C6—C7—C8	25.9 (3)	S1—C16—C17—N1	−2.5 (2)
C6—C7—C8—C9	176.15 (18)	C17—N1—C18—N2	179.14 (19)
C7—C8—C9—C10	174.4 (2)	C17—N1—C18—S1	−2.8 (2)
C7—C8—C9—C14	−8.0 (3)	C16—S1—C18—N2	179.25 (18)
C14—C9—C10—C11	0.1 (3)	C16—S1—C18—N1	1.12 (17)
C8—C9—C10—C11	177.91 (19)		

### Hydrogen-bond geometry (Å, °)

**Table:**

*D*—H···*A*	*D*—H	H···*A*	*D*···*A*	*D*—H···*A*
N2—H2A···O4^i^	0.88	2.07	2.926 (2)	163
N2—H2A···N1^i^	0.88	2.64	3.175 (2)	120
N2—H2B···O1S^ii^	0.88	2.05	2.872 (2)	154
O1S—H1S···O4	0.84	1.88	2.716 (2)	172

Symmetry codes: (i) *x*, −*y*+3/2, *z*+1/2; (ii) *x*, *y*, *z*+1.
